# Rachel Challice's Vow

**Published:** 1888-03-31

**Authors:** Lina Nevill

**Affiliations:** Author of "A Future on Trust," "Juno," &c.


### RACHEL CHALLICE'S VOW.

By LINA NEVILL, Author of "A Future on Trust," "Juno," &c.

CHAPTER XVII.—A LIFE'S WORK.

"Stands the mute sister, Patience, nothing loth,
And both supporting, does the work of both."—*Coleridge.*

THEY laid David to rest under the old, wide-spreading yew trees in Beth-gelert churchyard; beneath the trees which had seen so many generations of men and women pass away, and be brought to lie under their dark, protecting shadow, in the quiet Welsh resting-place.

Old Stephens had insisted on his son being buried at Beth-gelert in preference to Aberelwyn, and Esther and Rachel were willing to do as he liked in the matter. It made no difference to them. David was gone; and where his body lay was of little consequence compared to the sorrow for his loss.

Elias felt it differently. His old prejudices were unabated. Indeed, time and his lonely life, with such ample leisure for solitary musings, had rather strengthened his eccentricities than otherwise. He would rather his son were buried by any clergyman than by the Rev. John Jones, of Aberelwyn. Had cremation been easy to obtain, he would have flown to that means of disposing of his dead as an easy solution of his difficulty, and then have purchased the handsomest mourning urn he could find to receive his son's ashes, and probably have placed it side by side with his wife's monument in his back garden.

He wished now to do everything as handsomely as possible; but his enemy must not profit by his lavishness. To forgive to the uttermost farthing did not enter into Elias' creed. Proper pride and common fairness, as he termed his obstinacy, took the part with him of the spirit of the Sixth Commandment. It seemed a strange irony that, after all, the first way in which David should profit by his father's money should be for his funeral; and that Elias should be so soon called upon to fulfil the only promise he had held out to his son in the future—namely, that, if deserving, his funeral expenses should be defrayed.

"I didn't need to have altered my will," said the old man sadly to Rachel. "The poor lad will never have my money after all. It strikes me I was a bit too much in a hurry to take away what he'll never want."

Elias was very confidential to Rachel in those days before the funeral. He used to be continually coming in to know if there was anything he could do; anything they wanted fetched; or any message to be sent. Essie got quite used to the old man in his brown great coat, and would call out "gran," as Rachel had taught her to do, when she saw him coming, and boldly and unblushingly hold out her small hand for the pennies he never failed surreptitiously to bestow upon her.

He was always pressing bank notes and sovereigns on Rachel's acceptance; and would be deeply hurt if she refused them, saying she had not spent what he had last given.

"I don't want no stinting," he would say. "You must all have decent clothes," naming what he imagined was the one grand idea in every woman's mind, the salve for all woes, and the sole comfort for a sad heart. "And, mind, everything of the best for him, as well as you."

Only on one point was he obstinate, and deaf to all Rachel's entreaties.

He would not see Esther.

"I'll see her some day; and I'll walk with her at the funeral. But she always did rub me up the wrong way, and I don't want to feel set against her just now. My temper is apt to be a bit short now and again, as you, with your sharpness, may perhaps have noticed, Rachel Challice."

Rachel could not help smiling. The "shortness" of Mr. Stephens' temper had not escaped her observation. It required no particular sharpness to discover its existence.

Nell Endsleigh and her mother were very kind also.

They came every day to the cottage, and were profuse in offers of help, and really disappointed when Rachel assured them that the family wanted for nothing. Old Mr. Stephens was well off, and giving them everything they needed.

"If you will spare me a little longer that is the kindest thing you can do, Mrs. Endsleigh," said Rachel gratefully. "I don't want to leave my poor sister till things are a little more settled, and she is not fit to take entire charge of the children yet. I daresay in a week or so, when the funeral is over, she will be better, and then I will come back again."

"Of course we can spare you," cried Nell impetuously. "I miss you horribly all the same. Price's ways are not like yours, unfortunately, nor her temper either. Still, she can do everything for me, and I am getting so strong and well that a nurse will soon be a superfluity, so far as my health is concerned. Not that I am going to let you go till I am married," she went on decidedly. "Nell *Endsleigh* will want you so long as she lives. When she is no more, Nell *Austin* will always be only too glad to see you."

Nell was greatly taken with Essie. The child was the image of what her mother had been—fair, softly tinted, and delicately pretty, with winning little ways and gentle manners. Just as sweet, easily led, and unstable as Esther had been, and Esther's mother before her.

Essie, in her turn, worshipped Miss Endsleigh with an innocent childish adoration. She was never happier than when Nell would take off her rings and hang the glittering treasures on the tiny baby fingers.

"You will make her too vain, Miss Endsleigh," expostulated Rachel, who was somewhat of a Puritan in her distaste for ornaments; and who was by no means so pleased and amused as Nell by Essie's exhibition of her strong baby vanity. She had seen too much of the same failing in Essie's mother to wish for a repetition in the daughter. She knew also the sad history of her own dead mother; a knowledge that had been the key-note, in old days, of her tender watchful care of Esther.

The funeral day came. Nell had insisted on both the children coming to the hotel and spending the day there.

"It would be absurd to take them, they are too young for such things, and would only be in the way," she urged, when Rachel demurred, and said she could not think of putting Mrs. Endsleigh to so much trouble. "They can stay in Price's room when we do not want them, and that girl who is nursing baby can bring them."

Nell had her way, as she always did, in the end.

The children were dispatched to the hotel in the morning, under charge of the neighbour's girl; who considered the honour and glory of spending a day at the hotel, and being with the grand London ladies and gentlemen, an event to be remembered for ever in her quiet rural life.

It was a relief to get rid of the children, Rachel could not but confess it; though they were improved, and less spoilt and fractious, even in the few days that they had been under her management. Their mother was alternately weakly indulgent, and cross in her treatment of them; one moment letting the children do anything they liked, and the next slapping and scolding them.

Slowly the black procession wound down the straggling street. Half the village seemed to be attending the funeral, according to Welsh custom, to show respect to the dead and sympathy with the mourners.

Across the bridge then went, where foaming Colwyn loses itself in the broader bosom of the Glasslyn. Along the narrow road to the gate, and up the churchyard path.

Rachel remembered vividly the last time she had trodden that same path, when she had followed a bride and bridegroom along its windings nearly four years ago.

Now the bridegroom lay stiff and still under the dreary covering of the dark pall; and the bride hung, a weary, worn-out woman, on the arm of her father-in-law.

They laid David down under the old yew trees, and left him there; in the quiet resting-place where Glasslyn sings its sweet song to the dead, and the everlasting hills stand as grim untiring sentinels around.

Elias followed Esther and Rachel into the cottage on their return. "It is time we thought of the future," he said gruffly, trying to hide his emotion, which, however, nearly mastered him. "I'm going to do my duty by my son's widow and orphans, never fear. Now, my dear," he said, with an effort, turning towards Esther, who sat, a limp, black heap in an armchair by the fire, "which would suit you best, to live on here, or in any other house you liked, or come to me at Aberelwyn? You shall do just whichever you would like best; you've only to make up your mind, and everything shall be got ready for you and the children."

Esther looked piteously at Rachel, and then began to cry.

"Give us time to decide," said the eider girl coming to the rescue, as she saw how Esther's loud, uncontrolled grief irritated the old man, who so sternly kept his own under restraint.

"We have talked over no plans yet; and this is a question that cannot be settled in a hurry. Give Esther till to-morrow morning, and then we will let you know her decision."

"All right," said Stephens briefly, taking up his hat. "I suppose women do find their minds harder to make up than men. I remember Eliza Anne would never settle a thing without sleeping on it. And—and"— he went on looking towards Rachel—"the money won't go as I meant it to. I've given up the idea of that, though it was a praiseworthy and charitable idea too. It will all be for the children when I die; and I'm going to make you, Rachel Challice, and young James Lewis, of Pont-mawr, trustees."

He blurted out his statement as quickly as possible, rammed his hat on his head, and started off towards Aberelwyn.

"Oh, Rachel, I could never live with that dreadful old man—it would kill me," sobbed Esther. "I am so afraid of him; and he was so unkind and unjust to poor dear David and me. Let me stay on here with you, Rachel dear; I could be happy then, I think; and I never could be with Mr. Stephens at Aberelwyn.

Rachel was silent; a battle was going on in her mind.

It did seem hard that at every turn she must be sacrificed for Esther's good.

If Esther went to Aberelwyn there would be no necessity for her (Rachel) to give up the life she loved so dearly, and in which alone she found real peace and happiness.

Elias would be sufficient guardian for his daughter-in-law and grandchildren. He would manage everything; keep Esther from doing foolish things, and running into debt; and prevent her from utterly spoiling the children; and then Rachel would be free to live out her own life in her own chosen way.

It would not be exactly a happy home for Esther. Rachel could not, with all her sophistry, conceal this fact from herself. Elias' nature and Esther's were so completely antagonistic that even if there were not actual encounters, there would be always silent hostility.

Still, Esther had had her way. If things had turned out ill there was no one to blame. She had married David; he had died; and now she must bear the consequences, and make the best of the evil days that had fallen upon her.

It could not be necessary for Rachel to devote herself entirely, body and soul, to her service. She would always be ready to come if required; would see Esther and the children frequently, and assure herself of their welfare and happiness; and then, surely, she would have done all that any reasonable being could expect her to do. She herself would go out and work for her livelihood, while she would leave her sister in comparative idleness and luxury.

So Rachel wrestled, and tried, against her better judgment, to believe that her arguments were wise.

But the remembrance of her twice-repeated vow haunted her. The recollection would not be driven away.

"To shield and protect her at any cost to myself—never leave her so long as I can help her——"

Those words, spoken so long ago, dictated by her grandmother and repeated by herself, were as fresh and clear in her mind as when she had uttered them so many years ago.

She had vowed them over again, in effect, only a few nights since; and David Stephens had died satisfied of their fulfilment, sure of her truth and trustful in her faith.

The double promise to the dead rang in her ears, and refused to be silent.

"It is too hard! It is cruel and unjust!" she repeatedly wildly to herself. "Why should she have everything and I nothing?"

All night the battle raged, while the land was wrapped in sleep, and David Stephens lay resting well in his grave beside the rippling Glasslyn river, with the steep fir-clad hills beyond, and mighty Hebog casting his great shadow over the quiet village.

The calm moon looked in with its pure passionless radiance on the troubled girl, as wrong strove madly against right in that weak woman's frame.

The white stars, too, peeped in inquisitively, and watched breathlessly the issue of the battle.

When the moon and the stars waned the fight was over and the victory won. Inclination, with drooping tattered colours, fled before the unbroken lines of Duty. The sun rose up, and peace fell over Rachel's mind.

"I am going to live with my sister," she said calmly to old Stephens when he called in the morning to hear Esther's determination. "I know her and her ways, and she turns to me naturally for protection; and I love the children already. It is best to stay on here. You are too old to have all your ways upset, as they would be if Esther and the children went to Aberelwyn. You are accustomed to a quiet life, and their noise would worry you. It would not be fair, and you would soon be tired of having them."

"As you like," he replied, inwardly intensely relieved to find that the daughter-in-law he so cordially disliked, and whom he believed to be the true source of all David's misery and untimely death, was not to become an inmate of his home and a perpetual daily annoyance to him.

"I think you are as sensible as any woman *can* be; and I've never known you turn from doing what you thought was right, though your judgment mightn't always be wise, in my opinion. I'll see that none of you want. I won't shirk my share of the work, and what I say I always do. And I'll not forget you either, Rachel Challice, as you'll find out some day. You shall be no loser by your care of David's widow and orphans."

"I want no payment," she replied, smiling rather sadly. "I am doing it all for love."

No recompense the old man could make would ever be adequate to her sacrifice.

Only the sense of doing her duty, of fulfilling the last wishes of the man she had once loved, could reward her for her self-denial and steadfast loyalty.

Nell Endsleigh is Lady Austin now. She often comes down to Bedd-gelert in the autumn with her husband, and brings her boys and girls to see Nurse Challice, as she still fondly calls her.

Lady Austin, with her beautiful face, her riches, and her husband's position, is made much of in the great world, But she is just the same simple, warm-hearted, unaffected woman as she was in her young days, when Charley Austin loved her for her candour and bright good humour, and asked her, that summer afternoon at Hurlingham, to be his wife.

Her months of pain and sickness gave her nature just the qualities it lacked. Those stern tutors taught her gentleness and sympathy for others and patient enduring.

She always maintains that she should never have got well without Nurse Challice; and when Mrs. Endsleigh smiles and says, "For once, then, you will own that I was right," Lady Austin gives an unqualified assent, and humbly acknowledges herself to have been wrong; though her ladyship is no more fond of eating humble pie than she was in the old days when she was Nell Endsleigh.

And Rachel herself——

Time has changed her little. She is still a beautiful woman, with a sweet, calm face, and a quiet, dignified manner. Her sister leans implicitly on her, and can do nothing without her help and advice. Essie and Ray come to their aunt in every childish joy or sorrow, and obey her wishes without a murmur.

And she is happy——

In a calm, passionless way she is contented. She never repines over what she has lost, or mourns over the thought of a future that might have been hers.

She will go on in her patient, cheerful way, doing good to all with whom she comes in contact, till some day she too will lie down in Bedd-gelert churchyard, beneath the old yew trees, within sound of Glasslyn's rippling murmur, and sleep where David Stephens sleeps so soundly. And when that day comes she will be ready. Life does not hold her back. She is content to live; but death to her seems only the entrance gate to a happier existence, where all shadows and misunderstandings are made clear. We shall see plainly then what here seems a tangle of doubts and perplexities, for all will be revealed in the pure, searching light of a changeless, comprehensible eternity.

